# Supplementary material for: Long-read whole-genome analysis of human single cells
Source: Nat Commun. 2023 Aug 24;14:5164. doi: 10.1038/s41467-023-40898-3 (PMC10449900; doi:10.1038/s41467-023-40898-3)
Supplement: Supplementary file 1 — Supplementary Information [file 41467_2023_40898_MOESM1_ESM.pdf]

Supplementary Material: Long-read whole genome analysis of human single cells

Joanna Hård, Jeff E Mold, Jesper Eisfeldt, Christian Tellgren-Roth, Susana Häggqvist, Ignas Bunikis, Orlando Contreras-Lopez, Chen-Shan Chin, Jessica Nordlund, Carl-Johan Rubin, Lars Feuk, Jakob Michaëlsson, Adam Ameur

Table of contents:

Supplementary Figures 2
Supplementary Tables 5
Commands and parameters for PacBio single-cell variant analysis 14
  Alignment 14
  SNV calling 14
  SV calling (and multisample calling) 14
  Tandem repeat calling 14

Supplementary Figures

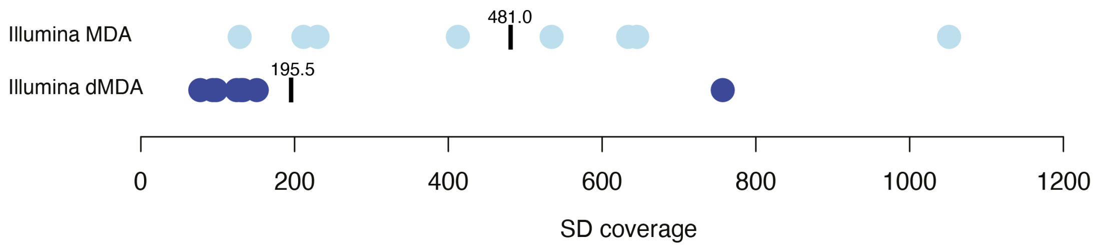

Figure S1. Standard deviation of coverage over the GRCh38 reference. The dots show the SD coverage value for Illumina single-cell MDA and dMDA samples, calculated by Qualimap. Average values are displayed by black vertical lines. The average SD coverage value is 2.46 times higher for MDA as compared to dMDA.

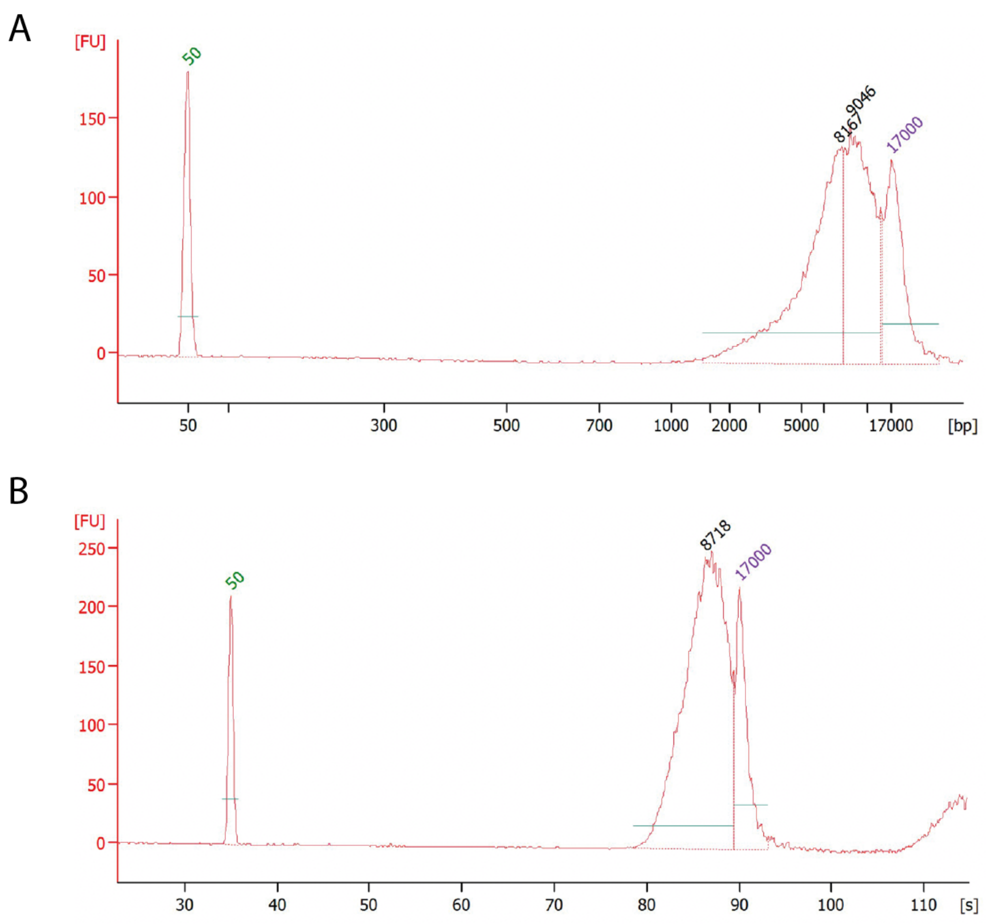

Figure S2. Fragment length distribution of a single-cell dMDA PacBio library. A) Bioanalyzer profile of SMRTbell library for T-cell A1 before size selection. **B)** Fragment length distribution of the same SMRTbell library after size selection using AMPure beads.

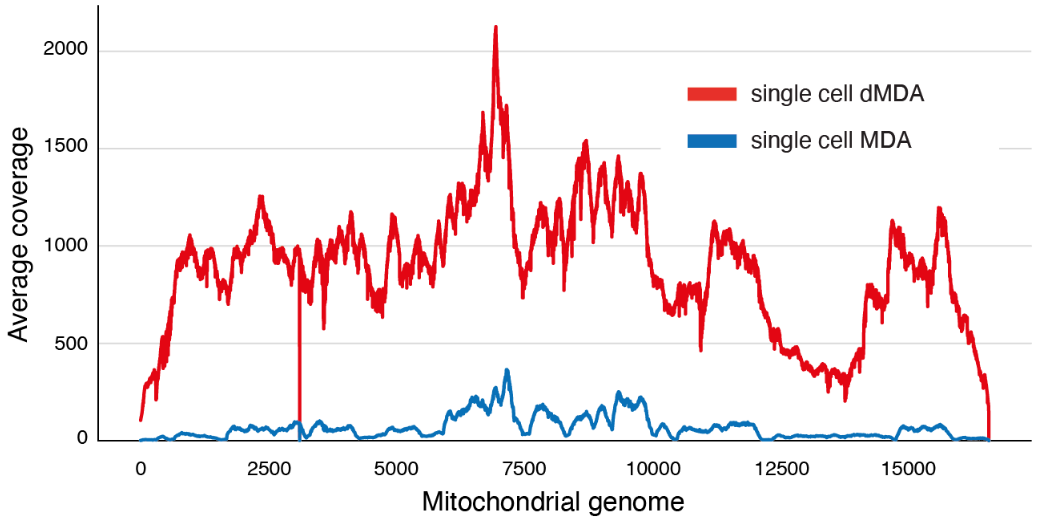

Figure S3. Average coverage over the mitochondrial genome. The two lines show the average coverage for the eight Illumina dMDA (red) and eight MDA (blue) single-cell samples.

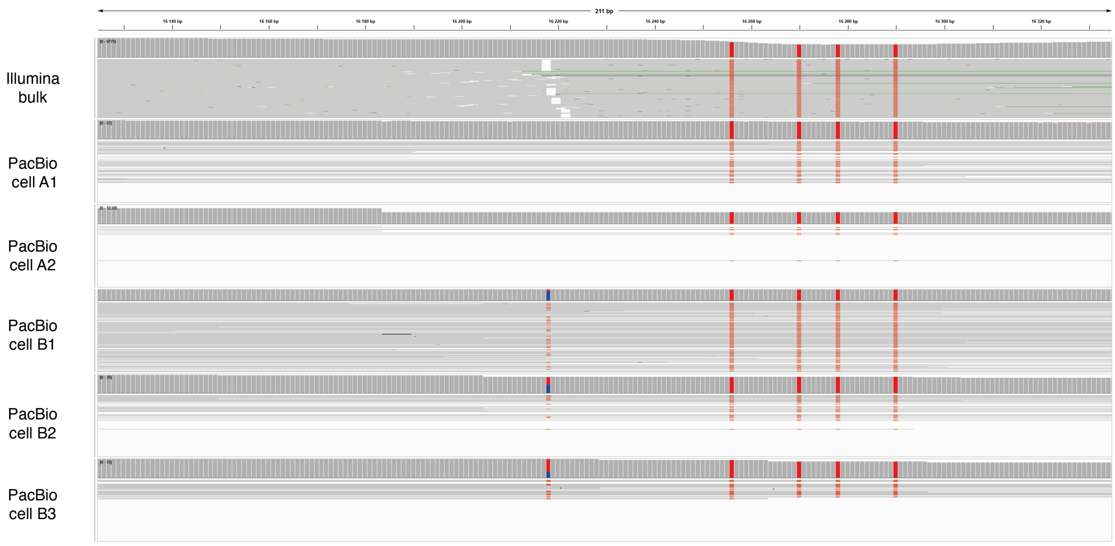

Figure S4. Mitochondrial heteroplasmy in T-cell B. IGV plot showing a 211 bp window surrounding position chrM:16,218 where the reference position is a C. In the PacBio data for T-cell B, 41-67% of the reads gave support for a T. In the bulk DNA and in T-cells A1 and A2, 0% of the reads gave support for the T substitution. The four red vertical lines correspond to variants in the mitochondria that are fixed in all three samples.

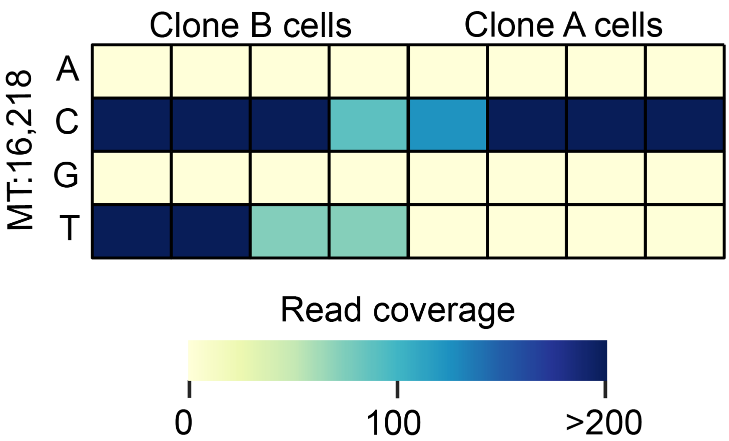

Figure S5. Validation of mitochondrial heteroplasmy in T-cell clone B. The heat map shows the distribution of different nucleotides (A, C, G and T) at position 16,218 in T-cell clones B (left) and clone A (right). Only data for the dMDA amplified samples are shown, since several of the MDA samples displayed low or no coverage at this position. All samples for clone B contain both the C and T bases. In the clone A samples only the base C is present.

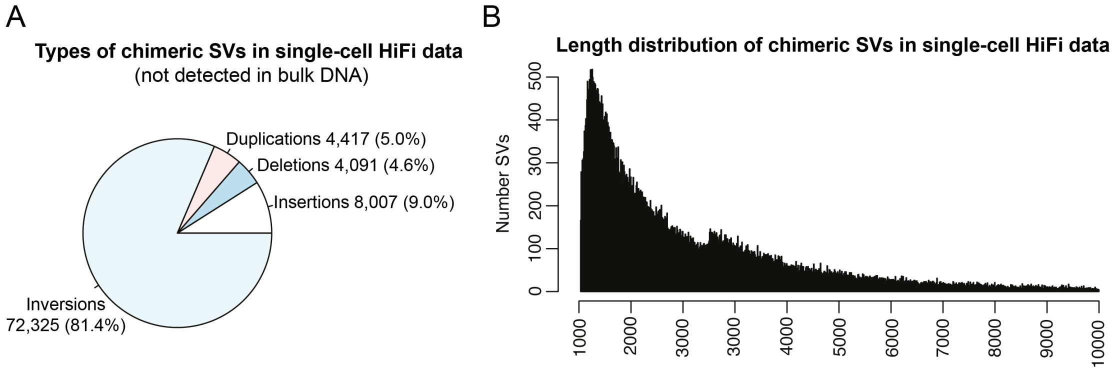

Figure S6. Classification of chimeric SVs detected in single-cell PacBio data. A) Pie chart showing the distribution of chimeric SVs in single cells, i.e. those only found in single cells but not in bulk PacBio HiFi data. A large majority of chimeric SVs are inversions (81.4% of all chimeric events). **B)** Length distribution of chimeric SVs.

Supplementary Tables

Table S1. Overview of Illumina WGS data generated in this project.

ID	Sample	Sample type	Total read pairs	Data amount (Gb)
Illumina_dMDA_A1	T-cell clone A	Single-cell dMDA	194979790	58.49394
Illumina_dMDA_A2	T-cell clone A	Single-cell dMDA	99366738	29.81002
Illumina_dMDA_A3	T-cell clone A	Single-cell dMDA	118075528	35.42266
Illumina_dMDA_A4	T-cell clone A	Single-cell dMDA	137472548	41.24176
Illumina_dMDA_B1	T-cell clone B	Single-cell dMDA	167428747	50.22862
Illumina_dMDA_B2	T-cell clone B	Single-cell dMDA	163128356	48.93851
Illumina_dMDA_B3	T-cell clone B	Single-cell dMDA	175128648	52.53859
Illumina_dMDA_B4	T-cell clone B	Single-cell dMDA	192217756	57.66533
Illumina_MDA_A1	T-cell clone A	Single-cell MDA	144803454	43.44104
Illumina_MDA_A2	T-cell clone A	Single-cell MDA	171922528	51.57676
Illumina_MDA_A3	T-cell clone A	Single-cell MDA	181849519	54.55486
Illumina_MDA_A4	T-cell clone A	Single-cell MDA	165570616	49.67118
Illumina_MDA_B1	T-cell clone B	Single-cell MDA	123531164	37.05935
Illumina_MDA_B2	T-cell clone B	Single-cell MDA	181450923	54.43528
Illumina_MDA_B3	T-cell clone B	Single-cell MDA	181372742	54.41182
Illumina_MDA_B4	T-cell clone B	Single-cell MDA	198105347	59.4316
Illumina_Bulk	PMBC Bulk DNA	PCR-Free WGS	416647211	124.9942

Table S2. Statistics for Illumina WGS datasets after random downsampling.

Sample type	T-cell clone	Downsampled reads	0x coverage (%)[a]	5-20x coverage (%)[a]
Single-cell dMDA	A (A1)	99976338	84.323	3.442
Single-cell dMDA	A (A2)	99366738	77.627	5.627
Single-cell dMDA	A (A3)	99999079	78.033	5.579
Single-cell dMDA	A (A4)	99994062	49.516	19.649
Single-cell dMDA	B (B1)	99983362	54.081	17.350
Single-cell dMDA	B (B2)	99990842	70.670	9.294
Single-cell dMDA	B (B3)	99996499	50.865	19.282
Single-cell dMDA	B (B4)	99987004	64.442	11.810
Single-cell MDA	A (A1)	99986588	66.993	6.780
Single-cell MDA	A (A2)	99992151	81.254	5.273
Single-cell MDA	A (A3)	100000818	83.715	4.369
Single-cell MDA	A (A4)	99985140	81.663	5.157
Single-cell MDA	B (B1)	100000534	72.341	4.941
Single-cell MDA	B (B2)	99998491	62.896	10.812
Single-cell MDA	B (B3)	99987332	68.818	10.040
Single-cell MDA	B (B4)	99980732	94.981	1.250
PCR-Free WGS	Bulk	100002110	9.243	89.212

[a] Percent of bases in the GRCh38 reference that has 0x or 5-20x coverage. The total size of GRCh38 is 3,088,286,401 bp.

Table S3. Coverage statistics for Illumina single-cell data.

Sample type	T-cell clone	Mean coverage [a]	SD coverage[a]
Single-cell dMDA	A (A1)	17.3245	756.7235
Single-cell dMDA	A (A2)	9.0343	98.1475
Single-cell dMDA	A (A3)	10.8345	150.8059
Single-cell dMDA	A (A4)	12.7484	77.2338
Single-cell dMDA	B (B1)	15.5136	124.4437
Single-cell dMDA	B (B2)	15.0295	130.4498
Single-cell dMDA	B (B3)	16.2603	93.2722
Single-cell dMDA	B (B4)	17.8	132.8106
Single-cell MDA	A (A1)	13.4643	412.4355
Single-cell MDA	A (A2)	15.9004	1051.4551
Single-cell MDA	A (A3)	16.923	211.6692
Single-cell MDA	A (A4)	15.389	230.0376
Single-cell MDA	B (B1)	11.4927	128.5143
Single-cell MDA	B (B2)	16.8636	645.7101
Single-cell MDA	B (B3)	16.9095	533.9986
Single-cell MDA	B (B4)	18.4422	634.1024

[a] Coverage statistics (mean and standard deviation) was calculated using Qualimap v2.1.1. The total size of GRCh38 is 3,088,286,401 bp.

Table S4. Overview of PacBio WGS data generated for the five single cells and for the bulk DNA sample.

Semple name	Sample	Sample type	Total reads	Data amount
PacBio single-cell A1	T-cell clone A	Single-cell dMDA	2,750,802	19.9 Gb
PacBio single-cell A2	T-cell clone A	Single-cell dMDA	1,561,444	15.3 Gb
PacBio single-cell B1	T-cell clone B	Single-cell dMDA	2,547,184	20.2 Gb
PacBio single-cell B2	T-cell clone B	Single-cell dMDA	1,040,050	10.0 Gb
PacBio single-cell B3	T-cell clone B	Single-cell dMDA	1,306,998	13.3 Gb
PacBio bulk	PMBC Bulk DNA	HiFi WGS	5,509,497	97.3 Gb

Table S5. PacBio Sequel II run statistics and alignment results for five human single T-cells obtained from the clones A and B

	**Clone A**		**Clone B**		
	**Single-cell A1**	**Single-cell A2**	**Single-cell B1**	**Single-cell B2**	**Single-cell B3**
≥ Q20 reads	2,750,802	1,561,444	2,547,184	1,040,050	1,306,998
≥ Q20 yield (bp)	19,880,131,345	15,332,739,249	20,169,954,798	10,010,164,105	13,331,286,206
≥ Q20 read length (mean, bp)	7,227	9,819	7,918	9,624	10,199
≥ Q20 read quality (median)	Q36	Q35	Q36	Q33	Q35
Number aligned reads	2,739,035 (99.57%)	1,557,468 (99.74%)	2,517,588 (98.83%)	1,036,841 (99.69%)	1,302,581 (99.66%)
Number of alignments	6,508,237	5,346,280	6,235,924	2,713,013	3,711,537
Aligned read mean concordance	99.18%	99.10%	99.11%	99.13%	98.91%
Aligned read length (mean)	2,994	2,785	3,149	3,620	3,525
Aligned read length N50	5,429	4,821	6,476	7,391	6,539
Aligned read length 95%	8,691	8,867	9,739	11,636	11,387
Aligned read length Max	43,386	41,038	48,731	51,021	54,566
Mean coverage	6	5	6	3	4
Covered bases[a]	39.60%	26.95%	27.71%	32.98%	28.86%

[a] The size of the GRCh38 reference genome used for these calculations: 3,088,286,401 bp

Table S6. Overview of SNVs detected in Illumina and PacBio single-cell WGS data

Sample type	T-cell	Total SNVs	Not found in bulk DNA	Found in bulk DNA[a]
Illumina dMDA	A (A1)	407683	199999	207684
Illumina dMDA	A (A2)	838786	224249	614537
Illumina dMDA	A (A3)	604022	217081	386941
Illumina dMDA	A (A4)	2098281	344340	1753941
Illumina dMDA	B (B1)	1927828	322682	1605146
Illumina dMDA	B (B2)	1166063	249078	916985
Illumina dMDA	B (B3)	2132715	359900	1772815
Illumina dMDA	B (B4)	1562806	347843	1214963
Illumina MDA	A (A1)	1688193	328200	1359993
Illumina MDA	A (A2)	886840	227187	659653
Illumina MDA	A (A3)	791362	208210	583152
Illumina MDA	A (A4)	850374	234589	615785
Illumina MDA	B (B1)	1469848	278451	1191397
Illumina MDA	B (B2)	1895984	341829	1554155
Illumina MDA	B (B3)	1567015	345818	1221197
Illumina MDA	B (B4)	336174	117117	219057
PacBio dMDA	A (A1)	1321172	177493	1143679
PacBio dMDA	A (A2)	892445	172484	719961
PacBio dMDA	B (B1)	944892	136726	808166
PacBio dMDA	B (B2)	1008831	108319	900512
PacBio dMDA	B (B3)	972394	137429	834965

[a] For Illumina data, the comparison was done to Illumina bulk, for PacBio to PacBio HiFi bulk

Table S7. Precision and sensitivity for SNV calls in single cell Illumina and PacBio HiFi data. The true positive SNV calls are defined as those detected in analysis of bulk data from the same sequencing technology.

Sample type	T-cell	Total SNVs	True SNVs[a]	Precision	Sensitivity
Illumina dMDA	A (A1)	407683	207684	0.5094252	0.04695395
Illumina dMDA	A (A2)	838786	614537	0.7326505	0.1389368
Illumina dMDA	A (A3)	604022	386941	0.6406075	0.08748103
Illumina dMDA	A (A4)	2098281	1753941	0.8358942	0.3965373
Illumina dMDA	B (B1)	1927828	1605146	0.8326189	0.3628972
Illumina dMDA	B (B2)	1166063	916985	0.786394	0.2073153
Illumina dMDA	B (B3)	2132715	1772815	0.831248	0.4008045
Illumina dMDA	B (B4)	1562806	1214963	0.7774241	0.2746832
Illumina MDA	A (A1)	1688193	1359993	0.8055909	0.3074722
Illumina MDA	A (A2)	886840	659653	0.7438241	0.1491367
Illumina MDA	A (A3)	791362	583152	0.7368966	0.1318411
Illumina MDA	A (A4)	850374	615785	0.7241343	0.1392189
Illumina MDA	B (B1)	1469848	1191397	0.810558	0.2693554
Illumina MDA	B (B2)	1895984	1554155	0.8197089	0.351369
Illumina MDA	B (B3)	1567015	1221197	0.7793142	0.2760927
Illumina MDA	B (B4)	336174	219057	0.6516179	0.0495252
PacBio dMDA	A (A1)	1321172	1143679	0.8656549	0.2178097
PacBio dMDA	A (A2)	892445	719961	0.8067287	0.1371141
PacBio dMDA	B (B1)	944892	808166	0.8552999	0.1539124
PacBio dMDA	B (B2)	1008831	900512	0.8926292	0.1714994
PacBio dMDA	B (B3)	972394	834965	0.8586694	0.1590162

[a] The SNVs detected in bulk sequencing are considered to be complete set of true positives. For the Illumina bulk the total number of SNVs is 4,423,142. For PacBio HiFi bulk the number is 5,250,817.

Table S8. Number of high-confidence SNVs in PacBio single cells that are overlapping with a "dark" region in clinically relevant regions of the human genome.

Sample	SNVs not in Illumina bulk	SNVs missing from Illumina bulk and in "dark" regions of clinical relevance
PacBio A1	108700	2715
PacBio A2	71655	1582
PacBio B1	91569	1641
PacBio B2	76694	1685
PacBio B3	87306	2513
Average	87184.8	2027.2
Total Unique SNVs	284082	6336

Table S9. Somatic SNVs detected in the two PacBio single-cells for clone A, but not in the PacBio bulk sample or clone B single cells.

	Chromosome	Position	Reference allele	Alternative allele
1	chr1	22628223	G	A
2	chr11	121519544	C	G
3	chr2	180266451	C	A
4	chr4	117567808	C	G
5	chr5	437214	G	A
6	chr8	36368389	C	T
7	chr8	48619816	C	T

Table S10. Somatic SNVs detected in at least two PacBio single-cells for clone B, but not in the PacBio bulk sample or clone A single cells.

	Chromosome	Position	Reference allele	Alternative allele
1	chr1	113193054	A	G
2	chr10	34415480	T	G
3	chr11	75164286	G	A
4	chr11	78106030	G	A
5	chr11	105534490	C	T
6	chr12	124661965	G	C
7	chr13	85887230	T	C
8	chr15	74233006	G	T
9	chr16	71091898	A	G
10	chr18	37520501	G	T
11	chr2	48617691	C	T
12	chr3	151588052	A	G
13	chr4	474440	C	T
14	chr4	170294389	T	G
15	chr5	98938587	T	G
16	chr6	165180217	A	T
17	chr7	154618000	C	T
18	chr8	23441489	C	T
19	chr8	76709056	G	A
20	chr9	108593173	G	C

Table S11. Precision and sensitivity for SV calls in PacBio single-cell HiFi data. The true positive SV calls are those detected in bulk PacBio HiFi data.

Sample type	T-cell	Total SVs	True SVs[a]	Precision	Sensitivity
PacBio dMDA	A (A1)	37001	6986	0.1888057	0.2755058
PacBio dMDA	A (A2)	35573	4535	0.1274843	0.1788461
PacBio dMDA	B (B1)	22756	4833	0.2123835	0.1905983
PacBio dMDA	B (B2)	20565	5749	0.2795526	0.2267224
PacBio dMDA	B (B3)	26126	5262	0.2014086	0.2075167

[a] For PacBio HiFi bulk the number of SVs is 25,357.

Table S12. Precision and sensitivity for deletion (DEL) calls in PacBio single-cell HiFi data. The true positive DEL calls are those detected in bulk PacBio HiFi data.

Sample type	T-cell	Total DEL	True DEL[a]	Precision	Sensitivity
PacBio dMDA	A (A1)	4333	3175	0.7327487	0.2802542
PacBio dMDA	A (A2)	3915	2152	0.5496807	0.189955
PacBio dMDA	B (B1)	2859	2165	0.7572578	0.1911025
PacBio dMDA	B (B2)	3167	2658	0.8392801	0.2346191
PacBio dMDA	B (B3)	3190	2399	0.7520376	0.2117574

[a] For PacBio HiFi bulk the number of DEL calls is 11,329.

Table S13. Precision and sensitivity for insertion (INS) calls in PacBio single-cell HiFi data. The true positive INS calls are those detected in bulk PacBio HiFi data.

Sample type	T-cell	Total INS	True INS[a]	Precision	Sensitivity
PacBio dMDA	A (A1)	5018	3804	0.7580709	0.2719474
PacBio dMDA	A (A2)	5746	2380	0.4142012	0.1701458
PacBio dMDA	B (B1)	3409	2659	0.7799941	0.1900915
PacBio dMDA	B (B2)	4256	3081	0.7239192	0.2202602
PacBio dMDA	B (B3)	4749	2862	0.6026532	0.2046039

[a] For PacBio HiFi bulk the number of INS calls is 13,988.

Table S14. Precision and sensitivity for duplication (DUP) calls in PacBio single-cell HiFi data. The true positive DUP calls are those detected in bulk PacBio HiFi data.

Sample type	T-cell	Total DUP	True DUP[a]	Precision	Sensitivity
PacBio dMDA	A (A1)	1396	0	0	0
PacBio dMDA	A (A2)	1456	0	0	0
PacBio dMDA	B (B1)	638	1	0.001567398	0.125
PacBio dMDA	B (B2)	367	2	0.005449591	0.25
PacBio dMDA	B (B3)	656	1	0.00152439	0.125

[a] For PacBio HiFi bulk the number of DUP calls is 8.

Table S15. Precision and sensitivity for inversion (INV) calls in PacBio single-cell HiFi data. The true positive INV calls are those detected in bulk PacBio HiFi data.

Sample type	T-cell	Total INV	True INV[a]	Precision	Sensitivity
PacBio dMDA	A (A1)	26254	7	0.000266626	0.21875
PacBio dMDA	A (A2)	24456	3	0.0001226693	0.09375
PacBio dMDA	B (B1)	15850	8	0.0005047319	0.25
PacBio dMDA	B (B2)	12775	8	0.0006262231	0.25
PacBio dMDA	B (B3)	17531	0	0	0

[a] For PacBio HiFi bulk the number of INV calls is 32.

Table S16. True positive SVs found in Illumina single-cell data, i.e. overlapping with an SV detected in Illumina bulk WGS data.

Prep	Clone	Deletions	Tandem_Dup	Inversions	Insertions	BND	Total
dMDA	A (A1)	0	0	0	0	4	**4**
dMDA	A (A2)	33	5	1	15	25	**70**
dMDA	A (A3)	21	2	0	9	14	**44**
dMDA	A (A4)	352	26	5	144	97	**584**
dMDA	B (B1)	360	20	3	124	117	**580**
dMDA	B (B2)	170	10	3	63	59	**283**
dMDA	B (B3)	412	25	6	152	128	**674**
dMDA	B (B4)	228	17	2	73	29	**373**
MDA	A (A1)	19	3	0	11	21	**46**
MDA	A (A2)	9	1	3	3	10	**21**
MDA	A (A3)	21	0	1	11	12	**40**
MDA	A (A4)	14	1	0	8	14	**35**
MDA	B (B1)	3	1	0	2	4	**10**
MDA	B (B2)	63	7	0	19	42	**123**
MDA	B (B3)	56	2	0	17	21	**91**
MDA	B (B4)	0	0	0	1	4	**5**

Table S17. Number of tandem repeat elements in the single cells having same repeat size as was found in the bulk sample

Sample	T-cell clone	Number TRs having same size as in bulk
PacBio A1	A	6512
PacBio A2	A	3512
PacBio B1	B	4916
PacBio B2	B	4504
PacBio B3	B	4404

Table S18: *De novo* assembly results for the two PacBio T-cells A1 and B1

	**Single-cell A1**	**Single-cell B1**
**Assembly statistics:**		
Filtered CCS reads (bp)	8,794,585,174 (44.2%)	9,405,139,162 (46.6%)
Assembly size, primary (bp)	598,293,718	454,096,399
Assembly completeness[a]	19.4%	14.7%
Contig N50	34,883	41,528
Max contig size	206,875	578,275
Assembly size, alternative (bp)	44,706,740	36,132,542
Contig N50, alternative	18,969	20,976
Max contig size, alternative	79,718	94,865
**BUSCO gene models:**		
complete	1762 (12.8%)	1236 (9.0%)
duplicated	17 (0.1%)	14 (0.1%)
fragmented	58 (0.4%)	250 (1.8%)
Missing	11960 (86.8%)	12294 (89.2%)
**Mitochondrion:**	Complete	Complete

[a] The size of the GRCh38 reference genome used for these calculations: 3,088,286,401 bp

Table S19: Detailed genome quality assessment (QUAST results) for the two *de novo* assemblies of PacBio T-cells A1 and B1

	**Single-cell A1**	**Single-cell B1**
# contigs (>= 0 bp)	20396	13388
# contigs (>= 1000 bp)	20396	13388
# contigs (>= 5000 bp)	20377	13376
# contigs (>= 10000 bp)	19526	13002
# contigs (>= 25000 bp)	9433	7070
# contigs (>= 50000 bp)	2560	2409
Total length (>= 0 bp)	598293718	454096399
Total length (>= 1000 bp)	598293718	454096399
Total length (>= 5000 bp)	598208138	454044271
Total length (>= 10000 bp)	591069613	450916370
Total length (>= 25000 bp)	413360580	344617035
Total length (>= 50000 bp)	174854660	181689921
# contigs	20396	13388
Largest contig	206875	578275
Total length	598293718	454096399
Reference length	3088286401	3088286401
GC (%)	40.60	40.31
Reference GC (%)	40.87	40.87
N50	34883	41528
N75	22337	25469
L50	5549	3406
L75	10928	6910
# misassemblies	12653	8049
# misassembled contigs	9803	6240
Misassembled contigs length	271814904	203400840
# local misassemblies	2190	1395
# scaffold gap ext. mis.	0	0
# scaffold gap loc. mis.	0	0
# unaligned mis. contigs	20	14
# unaligned contigs	3 + 279 part	10 + 190 part
Unaligned length	1053685	890329
Genome fraction (%)	19.711	14.769
Duplication ratio	1.031	1.045
# N's per 100 kbp	0.00	0.00
# mismatches per 100 kbp	114.26	116.47
# indels per 100 kbp	60.70	55.99
Largest alignment	206571	372886
Total aligned length	596128171	452514593
NA50	32291	38049
NGA50	-	-
NA75	19714	22183
LA50	5865	3666
LA75	11806	7574

Commands and parameters for PacBio single-cell variant analysis

Alignment

```
## HiFi read alignment and sorting for one sample
minimap2 --MD -ax map-pb hg38.fa cellA1_pacbio_reads.fastq >
cellA1.sam

samtools sort cellA1.sam -o cellA1.bam

samtools index cellA1.bam
```

SNV calling

```
## SNV calling for one sample
run_deepvariant --model_type PACBIO --ref hg38.fa --reads cellA1.bam
--output_vcf cellA1_deepvariant.vcf --num_shards 16 &>
cellA1_deepvariant.log

## Filtering of SNV results
grep -w '^#\|chr[1-9]\|chr[1-2][0-9]\|chr[X]\|chr[Y]\|chr[M]\|CHROM'
cellA1_deepvariant.vcf | grep -v "RefCall" >
cellA1_deepvariant_filtered.vcf
```

SV calling (and multisample calling)

```
## SV calling on one sample
sniffles --input cellA1.bam --vcf cellA1_sniffles.vcf --snf
cellA1_sniffles.snf --tandem-repeats
human_GRCh38_no_alt_analysis_set.trf.bed

## Multisample SV calling
sniffles --input cellA1_sniffles.snf cellA2_sniffles.snf
cellB1_sniffles.snf cellB2_sniffles.snf cellB3_sniffles.snf
hifi_bulk_sniffles.snf --vcf multisample.vcf
```

Tandem repeat calling

```
## Alignment with LAST
last-train -P8 -Q0 hg38lastdb cellA1_pacbio_reads.fastq >
cellA1_last.par

lastal -P8 -p cellA1_last.par hg38lastdb cellA1_pacbio_reads.fastq |
last-split > cellA1_last.maf

## Tandem repeat genotyping
tandem-genotypes-master/tandem-genotypes -u 1 -g tandem-genotypes-
master/refFlat.txt tandem-genotypes-master/rmsk.txt cellA1_last.maf >
cellA1_tandem_genotypes_all.txt
```
