# Supplementary material for: Long-read whole-genome analysis of human single cells
Source: Nat Commun. 2023 Aug 24;14:5164. doi: 10.1038/s41467-023-40898-3 (PMC10449900; doi:10.1038/s41467-023-40898-3)
Supplement: Supplementary file 3 — Description of Additional Supplementary Files [file 41467_2023_40898_MOESM3_ESM.pdf]

Description of Additional Supplementary Files

File Name: Supplementary Data 1
Description: Quality control results for single-cell datasets.
