# Supplementary material for: Long-read whole-genome analysis of human single cells
Source: Nat Commun. 2023 Aug 24;14:5164. doi: 10.1038/s41467-023-40898-3 (PMC10449900; doi:10.1038/s41467-023-40898-3)
Supplement: Supplementary file 5 — Reporting Summary [file 41467_2023_40898_MOESM5_ESM.pdf]

nature portfolio

Corresponding author(s): Adam Ameur

Last updated by author(s): Jul 18, 2023

Reporting Summary

Nature Portfolio wishes to improve the reproducibility of the work that we publish. This form provides structure for consistency and transparency in reporting. For further information on Nature Portfolio policies, see our Editorial Policies and the Editorial Policy Checklist.

Statistics

For all statistical analyses, confirm that the following items are present in the figure legend, table legend, main text, or Methods section.

n/a	Confirmed	
☐	☒	The exact sample size (*n*) for each experimental group/condition, given as a discrete number and unit of measurement
☐	☒	A statement on whether measurements were taken from distinct samples or whether the same sample was measured repeatedly
☒	☐	The statistical test(s) used AND whether they are one- or two-sided<br>*Only common tests should be described solely by name; describe more complex techniques in the Methods section.*
☒	☐	A description of all covariates tested
☒	☐	A description of any assumptions or corrections, such as tests of normality and adjustment for multiple comparisons
☐	☒	A full description of the statistical parameters including central tendency (e.g. means) or other basic estimates (e.g. regression coefficient) AND variation (e.g. standard deviation) or associated estimates of uncertainty (e.g. confidence intervals)
☒	☐	For null hypothesis testing, the test statistic (e.g. *F*, *t*, *r*) with confidence intervals, effect sizes, degrees of freedom and *P* value noted<br>*Give P values as exact values whenever suitable.*
☒	☐	For Bayesian analysis, information on the choice of priors and Markov chain Monte Carlo settings
☒	☐	For hierarchical and complex designs, identification of the appropriate level for tests and full reporting of outcomes
☒	☐	Estimates of effect sizes (e.g. Cohen's *d*, Pearson's *r*), indicating how they were calculated

Our web collection on statistics for biologists contains articles on many of the points above.

Software and code

Policy information about availability of computer code

Data collection	SMRTLink v10.1, NovaSeq Control Software 1.6.0/RTA v3.4.4, bcl2fastq v2.20.0.422, CASAVA v1.6, MultiQC v1.12, Qualimap v2.2.1
Data analysis	BWA mem v0.7.17-r1188, Samtools v1.17, BEDtools v2.29.2, Picard v2.20.4, Bcftools v1.10, TIDDIT v2.11.0, Manta v1.6.0, SVDB v2.4.0, Circos v0.69.9, Minimap2 v2.24, DeepVariant v1.5.0, Sniffles2 v2.0.7, Tandem Genotypes v1.1.0, IGV v2.8.13, hifiasm v.0.7-dirty-r255, QUAST v5.2

For manuscripts utilizing custom algorithms or software that are central to the research but not yet described in published literature, software must be made available to editors and reviewers. We strongly encourage code deposition in a community repository (e.g. GitHub). See the Nature Portfolio guidelines for submitting code & software for further information.

Data

Policy information about availability of data

All manuscripts must include a data availability statement. This statement should provide the following information, where applicable:

- Accession codes, unique identifiers, or web links for publicly available datasets
- A description of any restrictions on data availability
- For clinical datasets or third party data, please ensure that the statement adheres to our policy

The sequencing data presented in this paper contains sensitive personal information and is protected by the General Data Protection Regulation (GDPR). The data is deposited on a secure Swedish server and is available through the DOI: 10.17044/scilifelab.22730684. The data is available with restricted access regulated by the

data access agreement of Karolinska Institutet, Sweden. For requests to access the data, contact datacentre@scilifelab.se. Response to requests from the SciLifeLab data centre are expected to within days.

Human research participants

Policy information about studies involving human research participants and Sex and Gender in Research.

Reporting on sex and gender	Only one individual was examined in this study. Covariate-relevant population characteristics of the human participant is not relevant for the study.
Population characteristics	Only one individual was examined in this study. Population characteristics is not relevant.
Recruitment	Only one individual was examined in this study. The study participant was recruited through local advertising at the University hospital, Karolinska Institutet, Sweden. Inclusion criteria included age over 18, not previously vaccinated agains Yellow Fever Virus, not pregnant.
Ethics oversight	Regional Ethical Review Board in Stockholm, Sweden

Note that full information on the approval of the study protocol must also be provided in the manuscript.

Field-specific reporting

Please select the one below that is the best fit for your research. If you are not sure, read the appropriate sections before making your selection.

☒ Life sciences ☐ Behavioural & social sciences ☐ Ecological, evolutionary & environmental sciences

For a reference copy of the document with all sections, see nature.com/documents/nr-reporting-summary-flat.pdf

Life sciences study design

All studies must disclose on these points even when the disclosure is negative.

Sample size	No sample size calculation was performed. Our aim was to examine at least three individual cells with each of the sequencing methods (Illumina MDA, Illumina dMDA and PacBio dMDA), since this the minimal number of replicates in many other biological studies such as gene expression assays. In the end we were able to generate data for 5 PacBio dMDA single cell samples and 8 single cells each for Illumina MDA and dMDA. We believe that this sample size is sufficient for this proof-of-principle study although more single-cells would need to be analyzed to fully characterize the somatic differences between T-cell clones.
Data exclusions	No data was excluded from the study
Replication	Several single-cell samples were run with the different sequencing methods (Illumina MDA, Illumina dMDA and PacBio dMDA), see above.
Randomization	Single-cell samples containing sufficient amount of amplified DNA for library preparation and sequencing were randomly allocated to the different sample groups (Illumina or PacBio).
Blinding	Blinding of the single-cell samples was not possible, since we had to keep track of which sequenced were sequenced using the different methods.

Reporting for specific materials, systems and methods

We require information from authors about some types of materials, experimental systems and methods used in many studies. Here, indicate whether each material, system or method listed is relevant to your study. If you are not sure if a list item applies to your research, read the appropriate section before selecting a response.

Materials & experimental systems

n/a	Involved in the study	
☐	☒	Antibodies
☒	☐	Eukaryotic cell lines
☒	☐	Palaeontology and archaeology
☒	☐	Animals and other organisms
☒	☐	Clinical data
☒	☐	Dual use research of concern

Methods

n/a	Involved in the study	
☒	☐	ChIP-seq
☐	☒	Flow cytometry
☒	☐	MRI-based neuroimaging

Antibodies

Antibodies used

anti-CD3-PE/Cy5 (clone UCHT1), anti-CD14-V500 (clone MϕP9), anti-CD19-V500 (clone HIB19) (all from BD Biosciences)

Validation

Describe the validation of each primary antibody for the species and application, noting any validation statements on the manufacturer's website, relevant citations, antibody profiles in online databases, or data provided in the manuscript.

Flow Cytometry

Plots

Confirm that:

☐ The axis labels state the marker and fluorochrome used (e.g. CD4-FITC).

☐ The axis scales are clearly visible. Include numbers along axes only for bottom left plot of group (a 'group' is an analysis of identical markers).

☐ All plots are contour plots with outliers or pseudocolor plots.

☐ A numerical value for number of cells or percentage (with statistics) is provided.

Methodology

Sample preparation

Peripheral blood was collected from a healthy human donor by venipuncture. The donor was previously vaccinated with the live, attenuated Yellow Fever Virus (YFV) vaccine (YFV-17D) as part of an ongoing study to investigate the dynamics of adaptive immunity to YFV vaccination. To expand CD8+ T cell clones from single YFV-specific memory CD8+ T-cells, mononuclear cells were isolated from peripheral blood by density centrifugation and were first stained with HLA-A2/ YFV(LLWNGPMAV)-dextramer FITC (Immudex, Denmark) for 15min at 4°C, followed by staining with anti-CD8a-BV570 (clone RPA-T8, Biolegend), anti-CD3-PE/Cy5 (clone UCHT1), anti-CD14-V500 (clone MϕP9), anti-CD19-V500 (clone HIB19) (all from BD Biosciences), and LIVE/DEAD™ Fixable Aqua Dead Cell Stain (ThermoFisher) for 20 min at 4°C. After washing, single live CD14-CD19-CD8+CD3+HLA-A2/YFV-dextramer+ cells were sorted directly into 96 well U-bottom plates containing 500ng/ml HLA-A2/YFV peptide (LLWNGPMAV), 40U/ml human recombinant IL-2, and 40.000 irradiated (25Gy) CD3-depleted autologous PBMCs in T-cell media (RPMI1640 with 10% heat-inactivated human AB sera, 1mM sodium pyruvate, 10mM Hepes, 50[illegible]M 2-mercaptoethanol, 1mM L-glutamine, 100U/ml penicillin and 50[illegible]g/ml streptomycin) and were cultured for 20 days. Every 7 days half of the media was replaced with fresh T-cell media containing 50U/ml IL-2, 500ng/ml peptide, and 40.000 irradiated CD3-depleted autologous PBMCs, and the wells were visually inspected for proliferation. Clonal expansions of single HLA-A2/YFV-specific CD8+ T-cells clones were confirmed by flow cytometry by using the same staining protocol as described above. Clones with a sufficient number of clonal progeny were subsequently cryopreserved in fetal bovine serum with 10% DMSO and stored in liquid nitrogen until sorting for DNA/RNA sequencing analysis. To isolate single cells from two selected YFV-specific CD8+ T cell clones (A and B), the clones were thawed, washed twice in RPMI1640 supplemented with 10% fetal bovine serum, and stained with the antibodies described above and index-sorted into 96 well PCR plates (Thermo Fisher) or a dMDA cartridge containing lysis buffer as described in the following sections.

Instrument

For sorting for cloning we used a BD Fusion equipped with 355nm, 405nm, 488nm, 563nm and 637nm lasers

Software

For sorting and collecting flow cytometry data DIVA software was used (BD Biosciences). Post acquisition analysis was performed using FLowJo v10 (BD).

Cell population abundance

The data is based on sorting of single cells into each well, and as such it is not possible to test for purity after sorting. However, as each HLA-A2/YFV-specific T cell is expanded in a separate well and all expanded T cells in each well have a unique TCR, we can safely assume that there was indeed only one founder cell sorted into each well.

Gating strategy

Describe the gating strategy used for all relevant experiments, specifying the preliminary FSC/SSC gates of the starting cell population, indicating where boundaries between "positive" and "negative" staining cell populations are defined.

☐ Tick this box to confirm that a figure exemplifying the gating strategy is provided in the Supplementary Information.
